# Oral Manifestations of Exudative Erythema Multiforme in a Patient with COVID-19

**DOI:** 10.1155/2021/1148945

**Published:** 2021-08-30

**Authors:** Zana Sllamniku Dalipi, Fatmir Dragidella, Donika Kastrati Dragidella

**Affiliations:** Department of Periodontology and Oral Medicine, University Dentistry Clinical Center of Kosovo, Medical Faculty, University of Prishtina, Street Lagjja e Spitalit p.n., 10000 Prishtina, Kosovo

## Abstract

Coronavirus disease 2019 (COVID-19), caused by severe acute respiratory syndrome coronavirus 2 (SARS-CoV-2), is a viral infection with multiorgan manifestations that may affect the oral mucosa. The full range of oral manifestations of COVID-19 are unknown, and there are limited reports describing the features of oral manifestations of COVID-19, including taste loss, oral lesions, and xerostomia. The aim of this study is to report a case of oral erythema multiforme (EM) manifesting as oral, lip, and skin lesions in a COVID-19 patient. The presence of oral lesions in the late stage of COVID-19 could be related to weak patient immunity or related therapies.

## 1. Introduction

Coronavirus disease 2019 (COVID-19) is an infectious respiratory illness caused by severe acute respiratory syndrome coronavirus 2 (SARS-CoV-2), which has caused high rates of infection in different countries [1] and has been declared a global health concern by the World Health Organization (WHO) [2]. The most common symptoms are fever, dyspnea, dry cough, myalgia, anorexia, sore throat, tremors, confusion, headache, nausea, vomiting, and diarrhea [3, 4]. Respiratory complications of SARS-CoV-2 infection can progress to severe acute respiratory syndrome, or additional complications that involve the kidneys, heart, central and peripheral nervous system, and gastrointestinal tract [5].

Additional less-reported manifestations of COVID-19 also afflict a subset of patients.

Several reports have documented various orofacial manifestations in COVID-19 patients, including oral ulcerative lesions, vesiculobullous lesions, xerostomia, and acute sialadenitis [6–8]. Additionally, infection with SARS-CoV-2 may result in dermatological manifestations, including maculopapular exanthem, papulovesicular rash, urticaria, painful acral reddish-purple papules, livedo reticularis, lesions, and petechiae [9].

Erythema multiforme (EM) is a rare acute mucocutaneous condition of the skin and mucous membranes that has a wide range of clinical manifestations that can be mild (EM minor, EM major), fulminant, or severe (Steven-Johnson syndrome); it can even result in toxic epidermal necrolysis (TEN) [10]. EM manifests as skin eruptions with or without oral or other mucous membrane lesions [11, 12] and can develop at any age, but it develops most frequently in young adults [13].

Multiple etiological factors have been implicated; however, infectious agents are the major cause of EM in approximately 90% of cases. Herpes simplex virus (HSV) 1 and 2 are the main triggers in young adults (>80% of cases), followed by Epstein-Barr virus (EBV) and *Mycoplasma pneumoniae* [14]. Other triggers of EM include drugs, including antibiotics such as penicillins, cephalosporins, macrolides, and sulfonamides; nonsteroidal anti-inflammatory drugs; anticonvulsants; and others [15].

Lesions begin as numerous demarcated red or pink macules that subsequently become papular, and the papules may become gradually enlarged, forming plaques several centimeters in diameter. The central portion of the papules or plaques gradually becomes a dark red or brown color or purpuric. Crusting or blistering sometimes occurs in the center of the lesions. The diagnosis of EM relies on clinical indicators, and bloodwork may reveal mild leukocytosis, neutropenia, or mild anemia. Electrolyte values may be altered if the patient is dehydrated [16].

Some diseases often considered in the differential diagnosis of EM include autoimmune bullous diseases, drug eruption, figurate erythema, lupus erythematosus, pityriasis rosea, polymorphic light eruption, Steven-Johnson syndrome, TEN, urticaria, urinary vasculitis, vasculitis, viral exanthems, and other hypersensitivity reactions [12, 17, 18]. Systemic illnesses, whether infectious, genetic, autoimmune, or neoplastic, may affect the mouth cavity, and the early identification of oral lesions is usually challenging, but it always results in an early diagnosis and therapeutic initiation, with an improved prognosis and treatment outcome [19].

The prognosis is mainly related to the affected body surface area. Healing can occur spontaneously within 2 to 3 weeks for EM minor and within 4 to 6 weeks for EM major [20].

Regarding treatment and management, topical treatments include antiseptics for bullous lesions, antiseptic mouthwashes, and anesthetics. Ocular involvement is managed by an ophthalmologist. Healing is promoted by the application of Vaseline (petroleum jelly) on the lips.

## 2. Case Presentation

In this paper, we present a case of EM in a 17-year-old male patient with oral lesions who was confirmed to be positive for SARS-CoV-2 by reverse transcriptase-polymerase chain reaction (RT-PCR) amplification of viral RNA from a nasal swab. He was diagnosed with COVID-19; he experienced fever, cough, headache, muscle pain, and loss of taste and smell. He was prescribed penicillin, acetaminophen, and the anticoagulant nadroparin. He was treated as an outpatient under quarantine.

Two weeks after diagnosis, the patient was referred to our clinic, the Department of Periodontology at the University Dentistry Clinical Center in Kosovo. The patient had several complaints, including severe pain in the mouth, poor appetite, inability to eat or speak, burning of the mouth, fatigue, and occasional bleeding from the mouth.

**Figure 1:**
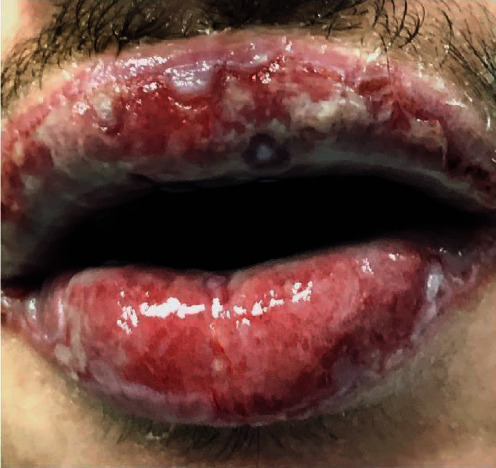
First visit to our clinic. Irregular ulcerations with whitish fibrin coating over vermilion-colored tissue.

**Figure 2:**
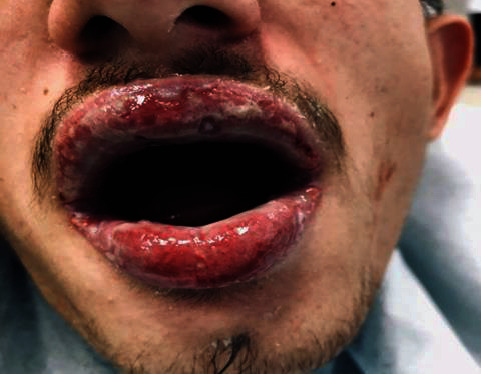
First visit to our clinic. Lesions extending to the oral mucosa.

**Figure 3:**
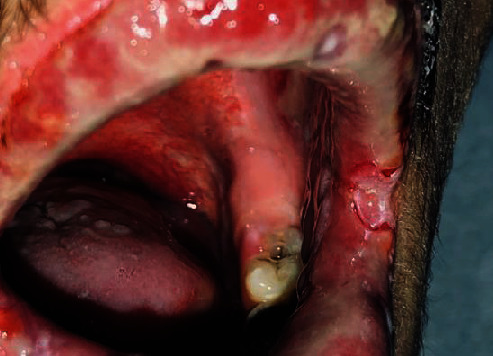
First visit to our clinic. Ulcerations on the left labial mucosa surrounded by a red ring and ulcerations on the left buccal mucosa.

**Figure 4:**
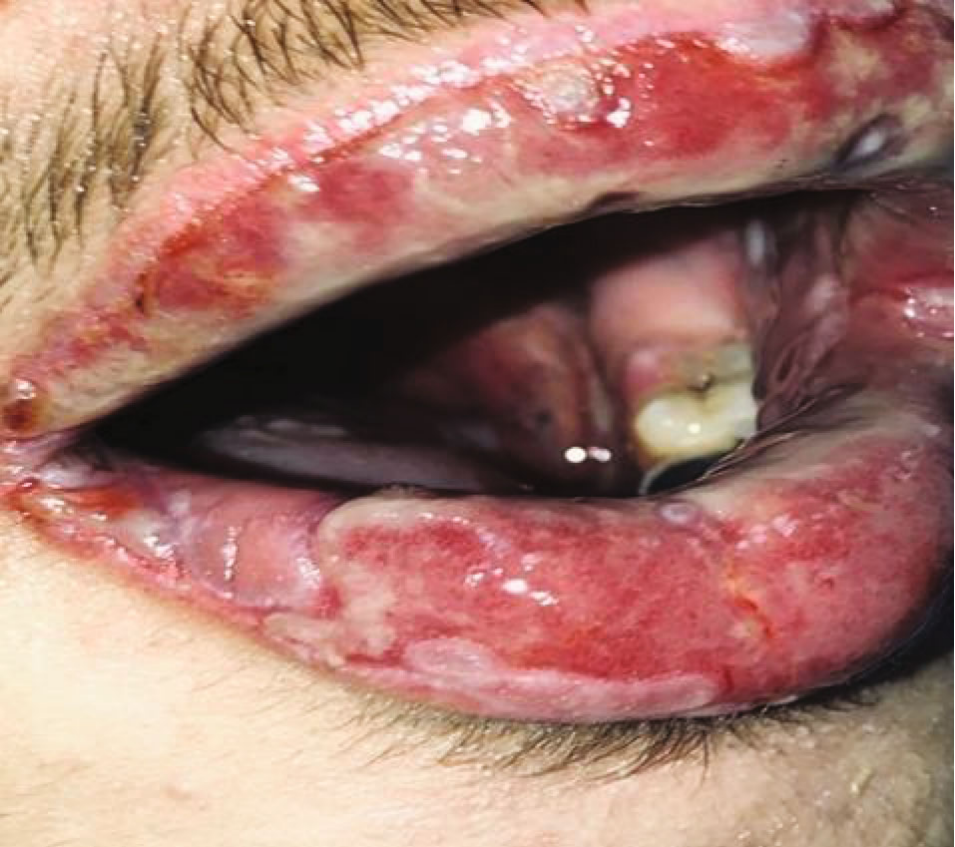
First visit to our clinic. Lip lesions with a tendency to bleed.

**Figure 5:**
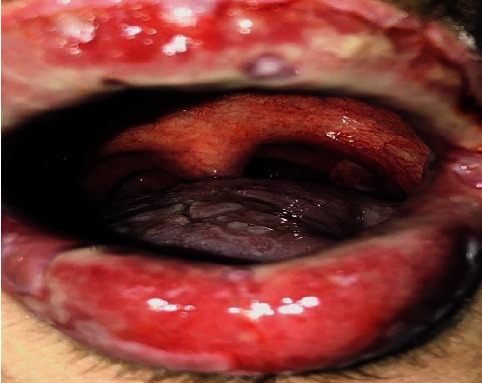
First visit to our clinic. Intraoral white coating on the tongue and redness of the isthmus faucium.

**Figure 6:**
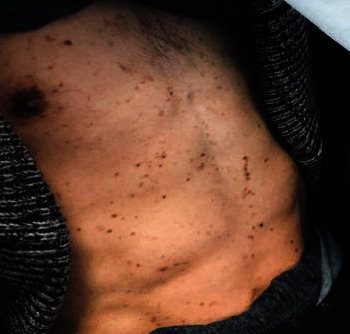
First visit to our clinic. Numerous dark red purpuric maculopapular lesions.

**Figure 7:**
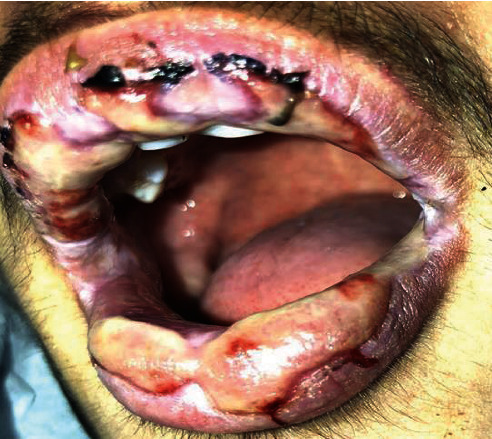
10 days after initial treatment with prednisone: bullae and crusts with vermilion-colored tissue.

**Figure 8:**
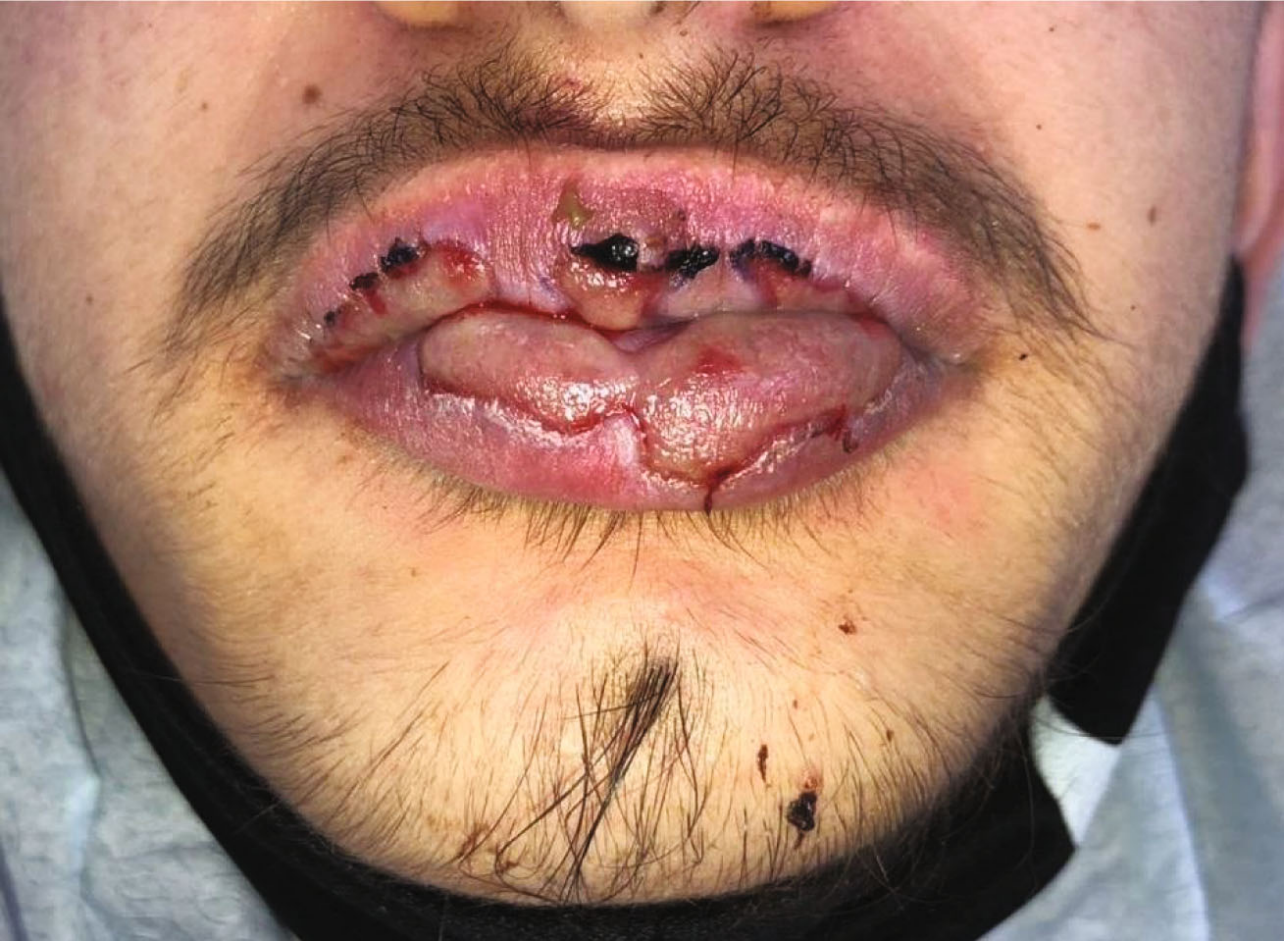
10 days after the first visit. Thick hemorrhagic crusts over the labial lesions and bullae.

Anamnestic and extraoral and intraoral physical examinations were performed at our clinic. The patient's lips and surrounding oral mucosa were vermilion in color. Bullous and erosive erythematous lesions covering the lips had caused severe erosion (Figures 1 and 2). There were vesiculobullous/macular lesions on the oral mucosa, and the clinical presentation consisted of EM-like lesions (Figures 3–5). At the time he presented at our clinic, pulmonary signs, such as cough and dyspnea, were absent, but numerous dark red, purpuric, irregular maculopapular lesions were present on the patient's abdomen (Figure 6). The patient had no complaints regarding the skin changes. Mucosal vesicles or bullae on the lips had ruptured and left the surfaces covered in thick white or yellow exudates, and ulcerations with bloody crusting were observed (Figures 7 and 8). The ulcerations were painful. The lesions were mainly associated with areas of poor oral hygiene. During the clinical examination, increased salivation was observed, which from time to time, according to the patient, was accompanied by blood.

Oral hygiene was very poor due to difficulties in oral care because of the pain associated with the mucosal lesions. The bilateral submandibular lymph nodes were enlarged and tender.

After he was diagnosed with COVID-19, his treatment was managed by an infectious disease specialist and included antibiotic therapy with penicillin and anticoagulant therapy with Fraxiparine solution for injection 0.4 mL (nadroparin calcium) (0.4 mL once per day for 7 days). On the day he presented to our clinic, the D-dimer level was 0.850 ng/mL FEU (reference: <0.500 ng/mL FEU) after 2 weeks, and the D-dimer level was within the normal range of 0.495 ng/mL FEU. The skin changes were reported 7 days after confirmation of COVID-19, and on the day he presented to the clinic due to oral changes, the skin changes were in the remission phase.

Laboratory examination revealed mildly increased in the white blood cell (WBC) count, C-reactive protein level, erythrocyte sedimentation rate, and D-dimer level. Leukocytes 11.51 (reference 10 ⋏/*μ*L), C-reactive protein 7.2 mg/L (reference < 5 mg/L), erythrocyte sedimentation rate 16/32 mm/h (reference 3-15 mm/h), and D-dimer level 0.850 *μ*g/mL FEU (reference < 0.500 *μ*g/mL FEU).

Sampling of the oral mucosa and dorsal surface of the tongue did not identify any bacterial or mycotic pathogens.

**Figure 9:**
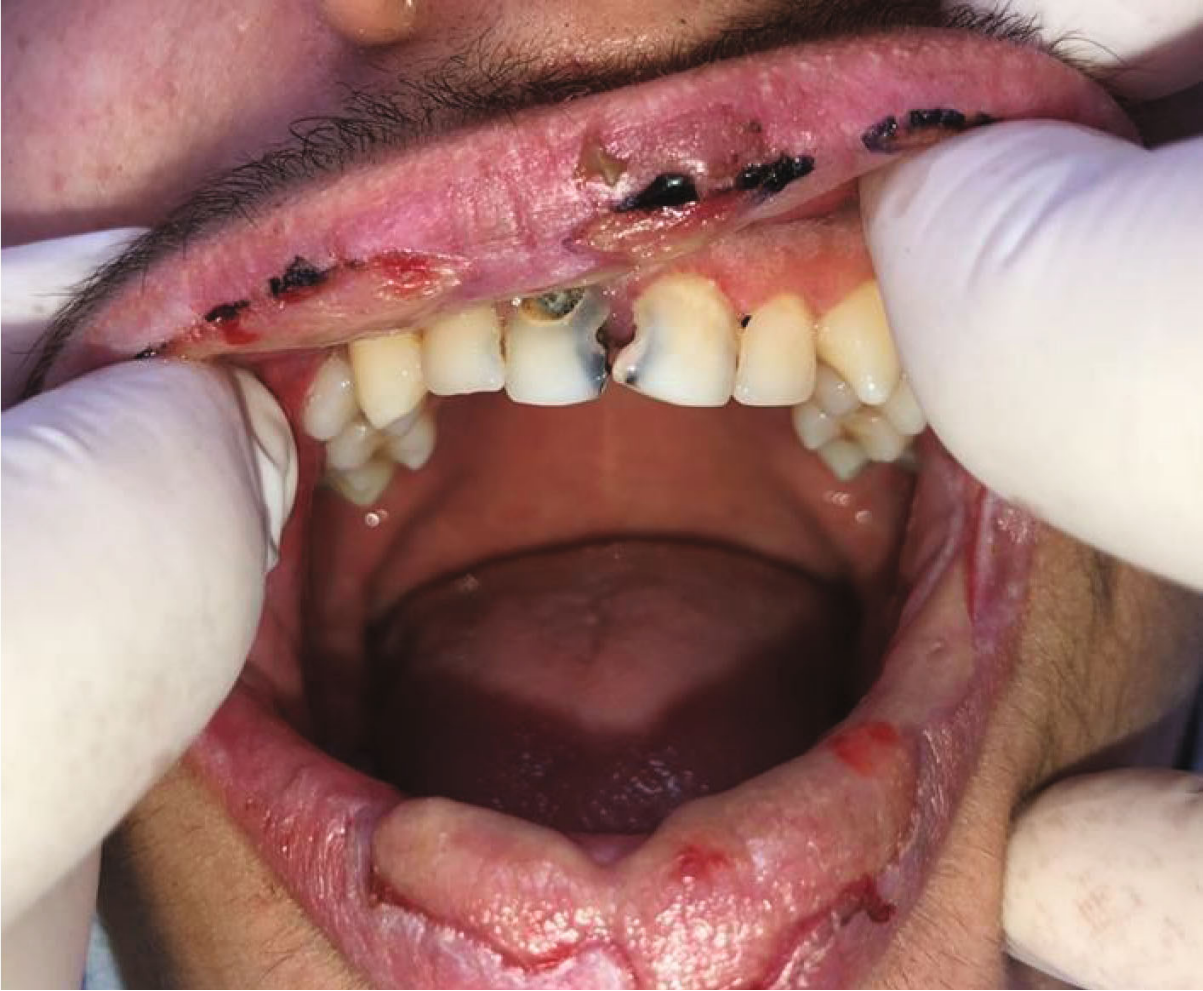
10 days after the first visit. Oral lesions only on the lips and labial mucosa.

**Figure 10:**
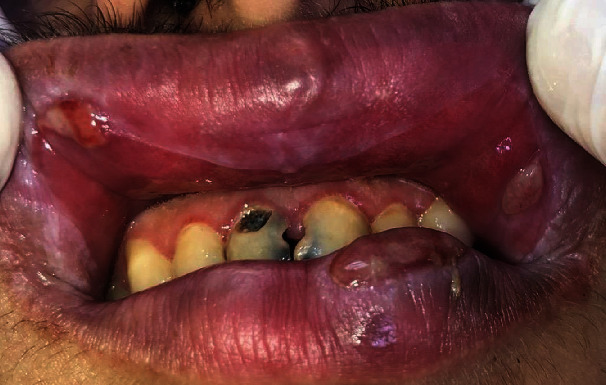
3 weeks after the first visit. Vesicles on the mucosa of the upper lip and bullae on the lower lip.

**Figure 11:**
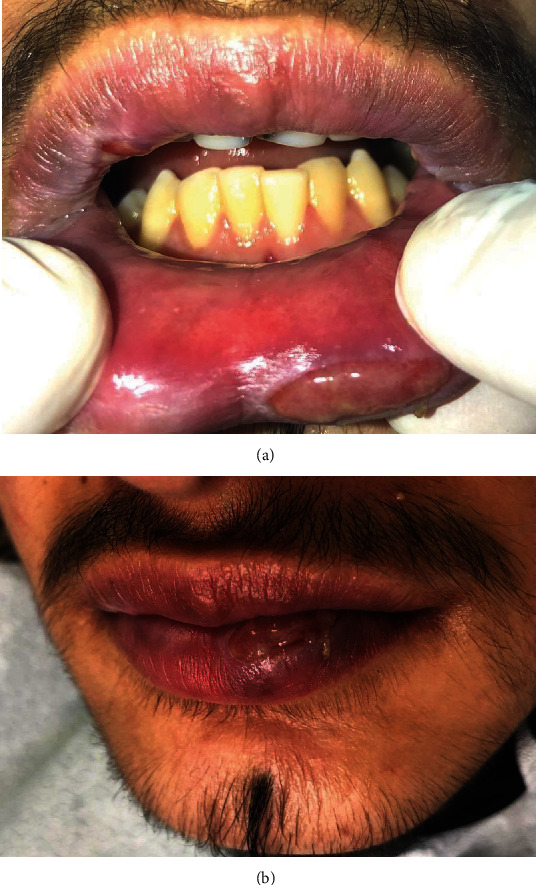
(a, b) 3 weeks after the first visit. Vermilion-colored upper lip without changes.

The patient received topical antiseptic treatments for the bullous lesions. The topical treatments were applied with wet gauze and an antiseptic mouthwash (0.2% chlorhexidine solution mouthwash twice per day for 14 days). Systemic corticosteroids, vitamins (C, B complex), and locally applied tablets (panthenol-calcium with pantothenic acid) were also prescribed to promote the epithelialization and regeneration of the epithelium of the oral mucosa. The patient received instruction regarding proper oral hygiene. The crusts on the lips were moistened with 0.9% sodium chloride saline solution until they could be removed without bleeding. Lip healing was promoted by the application of Vaseline (Figures 9–11).

**Figure 12:**
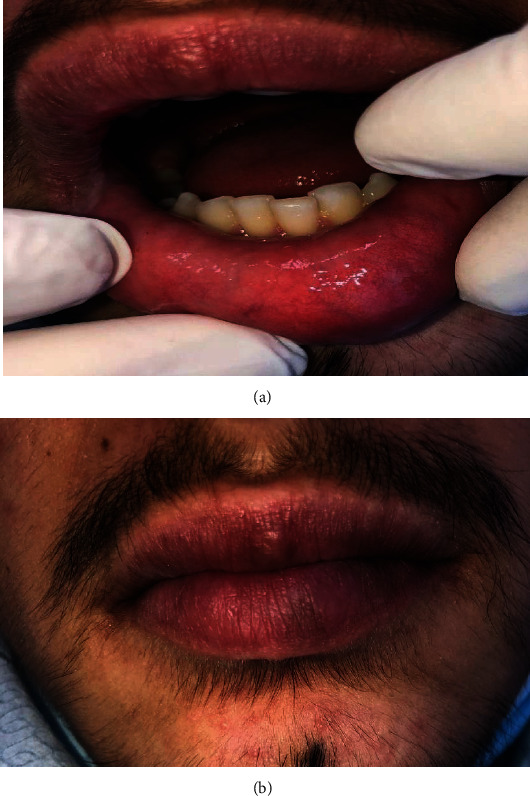
(a, b) 6 weeks after the first visit: without lesions on the lips and oral mucosa.

Complete remission of the oral manifestations was observed within approximately 3-4 weeks, and the vermilion discoloration resolved 5-6 weeks after initial admission to our department (Figure 12).

The patient provided written informed consent, and this case report was reviewed and approved by the Joint Ethics Committee of the University Dentistry Clinical Center of Kosovo.

## 3. Discussion

To our knowledge, this is the first COVID-19 case reported in Kosovo that involved changes in oral mucosa and skin.

Oral lesions are not directly caused by SARS-CoV-2 infection but are a secondary manifestation due to the host's immune response. Further studies are needed to evaluate whether these lesions are associated with the virus, the drugs used to treat infection, or any other conditions.

The oral mucosal epithelium is a complex barrier structure with a substantial number of functions; consequently, it can respond to various microorganisms and their toxic substances, mechanical impacts, and other exogenous factors [21]. Oral EM caused by drugs like penicillin is rare and reported to be less than 10% [22]. Although the first outbreak of drug-induced EM is limited to the oral mucosa, the following attack can result in more severe types of EM affecting their skin [23]. The possibility of EM being induced by penicillin was not considered as the patient had no history of adverse reaction associated with penicillin administration, the patient's lesions began in the skin and progressed to the oral mucosa, and the penicillin treatment had ended one week before being referred to the clinic.

Bapst et al. [24] reported the first case of EM in a pediatric patient with multisystem inflammatory syndrome in children (MIS-C) that was temporally related to COVID-19.

Oral involvement occurs because of the host inflammatory response in COVID-19 patients and is associated with morbidity and mortality outcomes [25].

The appearance of oral lesions in the early stages of COVID-19 might be an early indicator of peripheral thrombosis, indicating a likely progression to serious sickness. This implies that anticoagulant therapy should begin as soon as possible [26].

D-dimer levels are commonly elevated in patients infected with SARS-CoV-2. Significantly higher levels are found in those with critical illness and may be used as a prognostic marker for in-hospital mortality [27].

This case report could deepen the understanding of oral manifestations associated with COVID-19.

## 4. Conclusion

Clinical oral examination should be a standard part of the protocol for patients with confirmed SARS-CoV-2 infection. However, further studies are required to determine whether SARS-CoV-2 infection is the cause of, or a predisposing factor for, the development of oral symptoms and lesions.
